# Interpretation of LDH Values after Kidney Transplantation

**DOI:** 10.3390/jcm13020485

**Published:** 2024-01-16

**Authors:** Zoltán Sándor, Dorottya Katics, Ádám Varga, Károly Kalmár Nagy, Péter Szakály

**Affiliations:** Department of Surgery, Clinical Center, University of Pécs, 7624 Pécs, Hungary; dorottyakatics@gmail.com (D.K.); varga.adam@pte.hu (Á.V.);

**Keywords:** kidney transplantation, delayed graft function vs. acute rejection, elevated lactate dehydrogenase, early acute rejection diagnosis

## Abstract

Kidney transplantation is the gold-standard therapy for end-stage renal disease. However, in the early postoperative period following allograft kidney transplantation, insufficient graft function presents a diagnostic challenge to clinicians. Ischemic damage to the graft and/or an early autoimmune rejection may cause a decrease in function. Ischemic damage is a benign and transient condition, while acute immune rejection requires immediate therapy. A kidney graft ultrasound may produce a false negative result, and graft biopsy is invasive and slow to return results. Serum lactate dehydrogenase (LDH) is under examination as a possible tool for differential diagnosis between ischemic damage and immune rejection. Herein, we analyze the continuous lab results of four patients in the early post-transplantation period, showing patterns correlating with different clinical outcomes and prognoses. In our experience, a persistent elevated LDH accompanies ischemic damage. Immune rejection was, however, associated with a decrease in LDH. Hemodialysis was not a confounding factor, while packed red blood cell transfusion caused severe diagnostic problems.

## 1. Introduction

Chronic kidney disease (CKD) is defined as a persistent decrease in renal function lasting 3 months or more, and is a leading cause of morbidity and mortality worldwide. The expected age-related decline of renal function is exacerbated by primary kidney diseases, cardiovascular disease, diabetes, and several other risk factors that are globally prevalent. A 2016 meta-analysis found a worldwide prevalence of 13.4% for CKD, with moderate differences in the observed geographical regions [1]. The sum damage caused by these factors is generally considered irreversible, and patients are at risk of progressing to end-stage renal disease (ESRD). Globally, 759 pmp adults are receiving treatment for ESRD [2]. These patients are forced to use dialysis indefinitely, which severely reduces quality of life and is associated with high morbidity and mortality. A retrospective analysis of ESRD patients in Taiwan using renal replacement therapy showed a standardized mortality ratio of 7.3 among patients using peritoneal dialysis and 5.13 among patients using hemodialysis [3]. The alternative treatment and gold standard is kidney transplantation, which results in significantly better health outcomes with proper long-term care.

Following the implantation of the kidney allograft, the return of urine filtration will show significant differences between different patients. In an optimal case, a graft will immediately filter urine once circulation is restored, followed by 2–3 days of polyuria. In such cases, a decline in creatinine is usually apparent on the first postoperative day, and this process may hopefully continue uninterrupted until a stable balance is achieved. However, a common situation is the graft showing no function after implantation or showing decreasing function after the operation. This condition is called delayed graft function (DGF), and can be defined as the recipient needing dialysis in the early days after transplantation. Although this definition is the most widely utilized, several different definitions can be found in the literature; notably, a patient who did not require hemodialysis before transplantation would be unlikely to need dialysis even in cases of equivalent pathological processes in the graft.

Among other reasons, a decrease in graft function may be caused by ischemic damage suffered during the transplantation procedure or an early acute immune rejection of the graft.

Ischemic damage can be defined as the sum of all “trauma” suffered by the graft during retrieval, transport and implantation. Mainly caused by the loss of circulation and cooling during transport, this cellular damage mostly affects the tubules of the kidney. Based on this etiology, logic would dictate that these grafts would be dysfunctional from implantation, and would only show filtration once healing begins. However, in our practice, we routinely see urine production in the first 24–48 h even in cases of ischemic DGF. The kidney has extensive rejuvenation capabilities, and as such, ischemic tubular damage has an excellent prognosis, usually being transient and not requiring specific therapy. This is true even in cases of DGF lasting for weeks after transplantation.

Acute rejection (AR), on the other hand, is a process during which the immune system of the recipient begins an overt immunological process against the foreign tissue. Despite implantation being the first challenge to the recipient immune system, rejection is rarely seen immediately. Diligent crossmatching ensures that the recipient does not have pre-formed antibodies against the graft, while larger doses of immunosuppressive drugs administered perioperatively induce immunosuppression during the first exposure to foreign antigens. As a result of these techniques, hyper-acute rejection is a rare phenomenon; however, a slower acute rejection may develop in the days after implantation. AR can be divided into subcategories based on whether the reaction is mediated through T-cell infiltration and/or antibody binding to the blood vessels in the graft. Despite clinical differences, these subcategories may be grouped together for our current purposes. AR requires immediate steroid therapy, without which the process will aggravate and the graft will be irreversibly damaged.

These two conditions may also be concurrent; indeed, DGF caused by ischemia is itself a risk factor for acute rejection.

A timely diagnosis is thus needed to differentiate between the two etiologies in order to initiate therapy as needed. Currently used diagnostic methods are not precise and cause delays in the initiation of therapy. Urine output and serum creatinine have no differential diagnostic value with regard to these two conditions. Serum creatinine specifically is heavily influenced if the patient needs hemodialysis after transplantation. Dialysis will result in a sudden drop in serum creatinine levels, obscuring the native graft function. Doppler ultrasound (US) of the graft may provide a fast diagnosis of acute rejection, but is prone to false negative results. Testing the recipient for antibodies specific to the donor’s antigens (DSA) is a powerful diagnostic tool, but the scarcity of validated laboratories and the resulting turnaround time often mean that it is not available in acute settings for small-volume centers, notably our own clinic. A tissue biopsy of the graft has near perfect diagnostic value, but is an invasive procedure with risks and takes days to return results. Due to these limitations, a wide range of diagnostic tools are under consideration for use in differentiating these conditions.

Green et al. argue that serum lactate dehydrogenase (LDH) is a useful diagnostic marker, which allows us to differentiate graft ischemic damage and early acute rejection faster than the currently used methods. LDH is an abundant enzyme in the cells of several tissue types, among them the kidney tubules. Necrosis of these cells releases LDH into the bloodstream. A rise in serum LDH is, thus, a non-specific marker for the destruction of cells in the body. Necrosis of tubular cells is a hallmark of ischemic kidney damage, but is not significant in the early stages of acute rejection. Thus, a delay in graft function accompanied by an elevated serum LDH value would indicate ischemic damage to the graft. Meanwhile, signs of graft dysfunction accompanied by a normal LDH level would indicate acute rejection [4].

Truche et al. also support postoperative day 1 serum LDH as a clinically significant prognostic marker for DGF based on a series of 15 patients [5].

In contrast, Koyama et al. found that, in a series of 48 transplanted patients, LDH was not a reliable predictive marker for allograft damage. LDH levels varied widely and were mostly influenced by whether a graft from a living donor or a cadaveric allograft was implanted [6].

The reference value of serum LDH in a healthy population is 240–480 U/L at our institution, but it is important to note that different laboratories may set different reference values depending on the techniques used. This reference value is not to be taken for granted with our patients, as several factors affect the serum LDH level of an ESRD patient, even before implantation. Thus, we may encounter abnormal baseline serum LDH values even before the transplantation.

Al-Rubeaan et al. showed that the baseline serum LDH values in patients with ESRD can be up to 25% higher than in the normal population. Beyond the severity, the nature of the kidney disease (e.g., presence of proteinuria) also alters the serum LDH level [7].

Vaziri et al. argue that hemodialysis (HD) itself raises the baseline LDH levels by up to 15%. A single passage of blood through the dialysis machine significantly increases the LDH-2, LDH-3, and LDH-4 isozyme values. The observed isozyme pattern indicates the destruction of platelets though mechanical stress. However, an entire hemodialysis session raises the levels of the isozymes LDH-1 and LDH-5, which originate from granulocytes [8].

We did not find any literature regarding the effect of peritoneal dialysis on serum LDH levels. Because the mechanism of peritoneal dialysis does not assume significant cellular destruction, we believe it would not cause significant changes in baseline LDH values.

The kidney transplantation procedure itself causes significant LDH changes in the recipients during the early postoperative days. The long incision of the abdominal wall and stoppage of blood flow to the lower limb for around 45 min both cause the release of LDH. Currently, there is no viable method to differentiate between LDH originating from the graft and that from the recipient in the clinical setting.

## 2. Methods

Our case study presents the LDH patterns of four cadaver kidney transplant recipients in the early postoperative days, comparing patterns, complications, and outcomes. Each of these four patients experienced one of four major clinical outcomes possible after transplantation. An uncomplicated postoperative period, a patient with ischemic DGF, one with acute rejection, and one with both DGF and acute rejection were included.

Our program receives grafts from living donors and donation-after-brainstem-death (DBD) donors. As such, we do not receive grafts from donation-after-cardiac-death donors. In our practice, for induction immunosuppression, we administer 1080 mg of mycophenolic acid per os 6 h before the implantation and 500 mg of i.v. methylprednisolone when circulation is restored to the graft. Concurrent with the steroid bolus, we administer furosemide and mannitol i.v., with the exact dosing being operator-dependent. Per os tacrolimus (or cyclosporin) is given staring from the third postoperative day. Dosing of methylprednisolone is gradually reduced to a maintenance level of 16 mg Q 24 h, and is further reduced, if possible, in the months after transplantation.

From a surgical perspective, the kidney grafts are positioned in either the right or left iliac fossa, and the renal vessels are anastomosed to the external iliac vessels. The ureter is anastomosed to the top of the bladder, and an anti-reflux technique is used. In case of the patient suffering from a condition which makes the bladder unavailable for anastomosis, we construct an ileal conduit at the time of admittance to the kidney wait list. During the operation, a segment of the small bowel is separated and bypassed. The resulting pouch is anastomosed to the skin to function similarly to a bladder. During implantation, the graft is positioned upside down, and the ureter is anastomosed to this pouch. This situation is quite rare and only relevant because one of our presented cases needed to undergo this atypical implantation.

During transplantation, we routinely take an intraoperative biopsy of the graft. This is performed on the backbench before implantation. Although useful as a benchmark if later biopsies are needed, this is primarily performed as a measure of graft quality. In each case, these biopsies showed some level of ischemic damage to the tubules. This preservation damage is graded using the acute tubular necrosis grading, with “ATN I” being minimal damage and “ATN III” being massive damage.

After transplantation, we take creatinine and LDH values every morning during the hospital stay. The blood samples are taken from a central venous catheter during the whole stay. On our charts, day “0” indicates the preoperative lab test taken on admission before the operation. Day “1” blood samples are taken the morning after the operation, at 6–12 h postoperative. From day “2” onward, every measurement is taken at 24 h intervals.

In our practice, we diagnose DGF clinically according to the following:

- The patient’s urine output remains insufficient, creatinine levels remain high or rise, and US shows no definitive signs of rejection;
- OR a patient needs hemodialysis during postoperative care.

In case of clinical suspicion, we routinely use US to diagnose early acute rejection. In one of our cases, repeated US examinations did not find signs of rejection, and the diagnosis was based on a postoperative graft biopsy.

Important to note is our institution’s use of graft biopsy as a method of last resort, especially in the early postoperative period. The use of histological analysis as the go-to method of diagnosis is widespread throughout the world. Many centers advocate for its use at the earliest suspicion of acute rejection, and even repeat the procedure several times in order to assess changes in the graft tissue. Surveillance protocol graft biopsies, taken at predetermined intervals regardless of symptoms, have also been adopted at several centers. Racusen et al. recommend a low threshold for graft biopsy, coupled with surveillance biopsies of selected patients at 3–12 months postoperatively. The authors cite a low complication rate of 0.4–1% as the basis for liberal biopsy use [9]. As expected, this has been a boon to research using biopsy-proven rejection as a well-defined data point in statistical analysis.

However, we maintain a protocol of avoiding biopsy in the first 10 days after transplantation, as in the past, we have experienced higher complication rates than expected, as well as several cases of patients needing repeated biopsies due to insufficient glomeruli in the samples. This could be explained by inexperience, as, on average, less than one biopsy is performed every month. Centers with large patient volumes would see reduced complication rates in line with the cited article. In our research, we do not require biopsy-proven rejection for categorization; we use radiological signs or a response to therapy instead.

We do not use donor-specific antigens in the acute setting because of technical limitations specific to our institution. Our in-house laboratory is not validated to perform DSA testing, and samples need to be analyzed at a national center. Both the resulting turnaround time for the results and the associated costs have prohibited us from relying on DSA testing for quick diagnosis. As seen with one of our cases, DSA testing is routinely used during follow-up of transplanted patients.

In cases of clinical or US signs of AR, we begin bolus steroid therapy as soon as possible without waiting for further tests. Specifically, i.v. methylprednisolone 500 mg Q 24 h is used for at least 3 days (up to 5 days). The dose of the patient’s own immunosuppressants is not changed while undergoing anti-rejection therapy. In the case of rejection resistant to methylprednisolone, we escalate to the use of intravenous immune globulin (IVIG) and rabbit antithymocyte globulin (rATG). These more aggressive therapies are not standardized, but are reviewed daily based on clinical response, patient lymphocyte levels, and side effects.

## 3. Results

### 3.1. Uncomplicated Recovery

**Figure 1:**
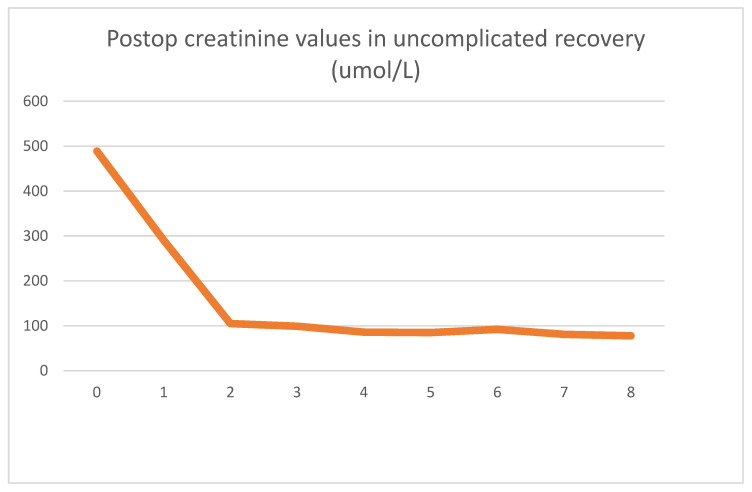
Daily creatinine values showing typical decrease and stabilization.

**Figure 2:**
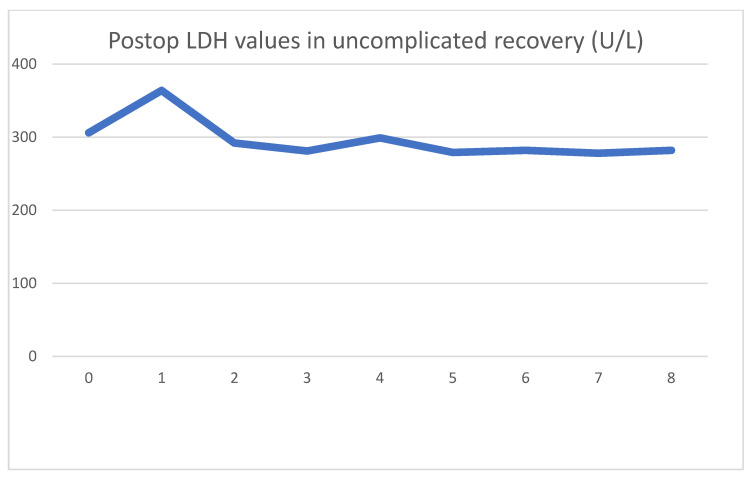
Daily LDH values spiking and then stabilizing at baseline.

Our first case presented is a 29-year-old male for whom a routine lab test revealed chronic renal disease. Follow-up tests indicated hereditary nephropathy, with a histology of focal segmental glomerulosclerosis. Concurrently, hypertrophic cardiomyopathy was diagnosed. On admission, he had spent 3 years in the hemodialysis program. Undergoing emergent surgery, he was implanted with a DBD cadaver kidney graft. Routine biopsy showed low-level preservation damage to the tubules (ATN I). The graft had already started filtering urine during surgery. The postoperative lab values observed were as follows Figure 1 and Figure 2.

Postoperative graft function was excellent, and no complications were seen. On day eight, he was discharged from the ward with a creatinine value of 78 umol/L. Two years of follow-up since the transplantation have been uneventful.

As seen clinically, within 2 days, his creatinine values dropped to close to the normal range and stabilized. After the operation, an LDH spike could be seen, which reversed in 24 h and returned to the patient’s baseline of 280 U/L. After the transient spike, the LDH values were stable throughout his stay. The absolute LDH values stayed within normal parameters.

### 3.2. DGF Secondary to Ischemic Damage

Our second presented case is a 42-year-old male with congenital omphalocele and exstrophy of the bladder. These congenital diseases necessitated that a uretero-sigmoidostomy be performed in childhood. Because of bilateral kidney stones and repeat episodes of infections, ESRD eventually developed. To remove the source of infection, the patient’s left kidney was removed even before referral to our center. Peritoneal dialysis was unsuccessful because of the previous abdominal surgeries, and hemodialysis was started instead. Before registration to the kidney wait list, his right kidney was also removed, as its size would have prohibited implantation, and the minimal function it provided was deemed an acceptable loss. His condition necessitated that an ileal conduit be prepared before transplantation, using the technique specified above. Though not a factor during admission, chronic anti-epileptic therapy was needed because of repeated seizures.

**Figure 3:**
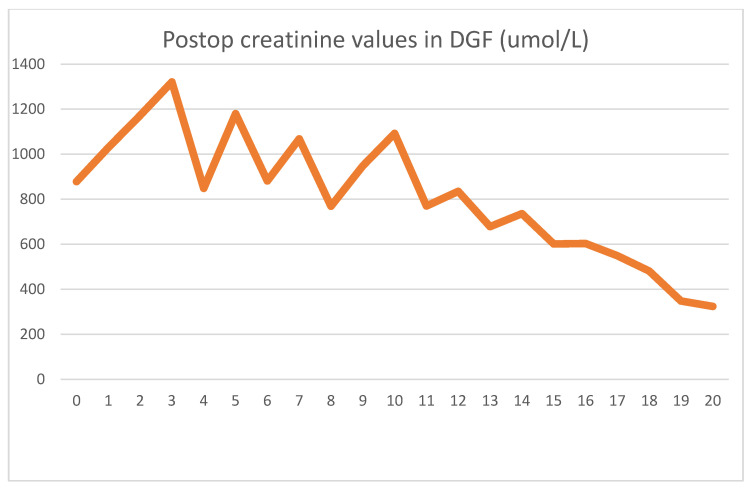
Daily creatinine values showing the effect of HD and gradual stabilization.

**Figure 4:**
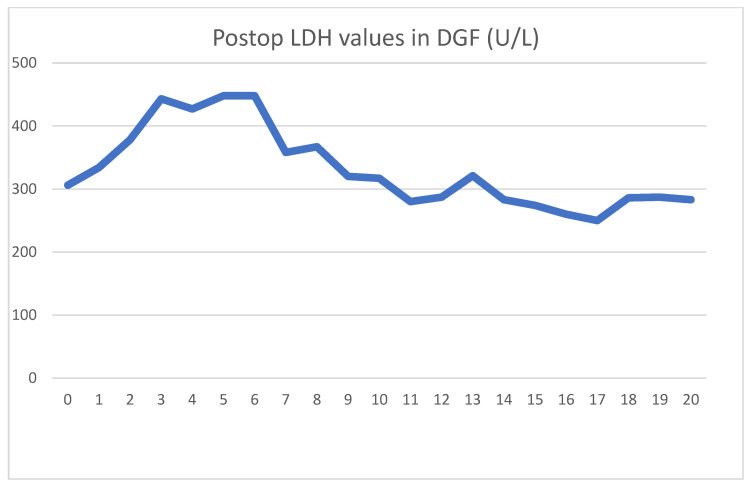
Daily LDH values showing typical LDH spike and stabilization. Blood transfusions on days 11 and 17 are followed by small increases.

The patient received a cadaver graft, which was implanted in an atypical upside-down position with the ureter anastomosed to the small bowel conduit. The biopsy showed slight preservation damage to the tubules (ATN I). Graft filtration did not begin during implantation, and postoperative lab values were as shown Figure 3 and Figure 4.

Until postoperative day 10, graft function was slow to start, and hemodialysis was needed every second day. Several US exams were performed, but showed no signs of acute rejection. Thus, we assumed that ischemic damage to the graft was the cause of the delayed function. The gradual increase in function without specific therapy supported our assumption, and after 10 days, hemodialysis was discontinued. Creatinine at discharge was 324 umol/L, which was still an abnormally high level.

Up until day 10, the effects of HD treatment dominated the serum creatinine values. After the patient had achieved adequate graft function, a slow decline could be seen, as expected.

Regarding serum LDH levels, it took 3 days to reach peak levels and another 3 days to achieve a meaningful decline. Contrarily to the literature, HD treatments did not affect LDH levels. During the transplantation, and on days 11 and 17, he received blood transfusions for anemia. Each of these were followed by transient LDH increases.

During the 3-year follow-up period, creatinine levels continued to slowly decline. At 3 months, a sterile collection, suspected to be a lymphocele, was detected next to the graft, which required drainage, first with a minimally invasive intervention technique. This proved insufficient, but drainage through surgical means was later successful. During this treatment, his graft function temporarily declined, and he again needed HD and red blood cell transfusion. With the clearing of the fluid collection, his graft function returned to normal levels. Apart from this, he was once admitted with signs of graft urological infection and successfully treated with intravenous antibiotics. Follow-up has since been uneventful.

### 3.3. Early Acute Rejection

Our third case is a 23-year-old female who suffered from therapy-resistant nephrotic syndrome with a histology of focal segmental glomerulosclerosis. At presentation, she was in a status of ESRD and had been using peritoneal dialysis for the last 2 years. Her anamnesis showed hypertension, hypercholesterinemia, and abducens nerve paresis.

**Figure 5:**
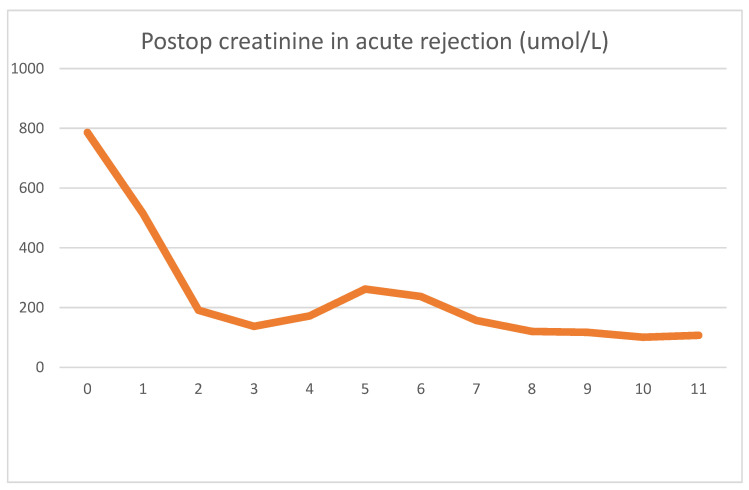
Daily creatinine value showing decrease, stabilization and an increase associated with acute rejection. Reversal of rejection is followed by stabilization of creatinine.

**Figure 6:**
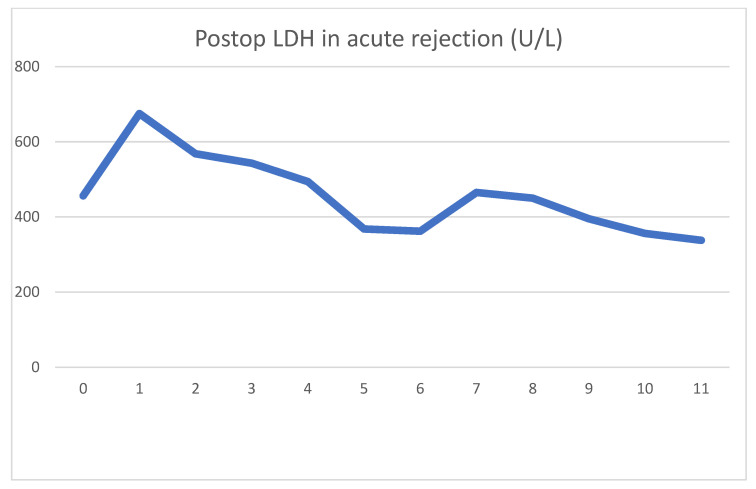
Daily LDH values first showing a typical spike. Acute rejection on day 4 is associated with a decrease in LDH.

During emergent surgery, she was implanted with a DBD cadaver graft. The biopsy showed minor preservation damage to the tubules (ATN I). The graft had already started producing urine during implantation. Postoperative lab values were as follows Figure 5 and Figure 6.

On day four, clinical signs of acute rejection were seen (decreased urine output, creatinine increase). US examination showed definitive signs of acute rejection. We used i.v. steroid therapy for 3 days per the standard protocol. After steroid therapy, her creatinine levels declined, and at emission, they were 107 umol/L. As was the case previously, we can see the serum LDH peak the day after surgery, which later decreased to normal values. The rejection diagnosed on day 4 was accompanied by an even sharper decrease in serum LDH. After steroid therapy, this decrease turned into a transient 2-day increase. The mechanism for this is uncertain, but tubular damage secondary to acute rejection may have been the cause. Steroid therapy itself is not associated with LDH increase. During this time, there were no other factors which could have caused the increase.

During the 18-month follow-up period, her creatinine levels rose steadily, and are currently above 500 umol/L. During this time, her US examinations were normal, and urine bacterial cultures were negative. Based on a negative donor-specific antigen, we assumed a relapse of her previous systemic disease, and different immunosuppressive regimes were used to try to stall the deterioration. Despite this, we were unsuccessful in improving her status; meanwhile, a graft biopsy showed signs of antibody-mediated chronic rejection. As was expected, her rejection progressed, and she is currently undergoing renal replacement therapy once again.

### 3.4. Combination of DGF and Acute Rejection

Our fourth case is a 43-year-old female who suffered from polycystic kidney disease. A central venous line was used for hemodialysis until she became septic and bacterial contamination was found on the end of the catheter. This caused endocarditis, which required open heart surgery to treat. A reoperation was needed to treat a pericardial hematoma in the early postoperative period. After several attempts, a Cimino arteriovenous access was achieved, and she used HD in the 3 months before transplantation. Her large polycystic left kidney caused mass symptoms, and was thus removed before referral to our center. A postoperative wound hernia was repaired at a later date.

**Figure 7:**
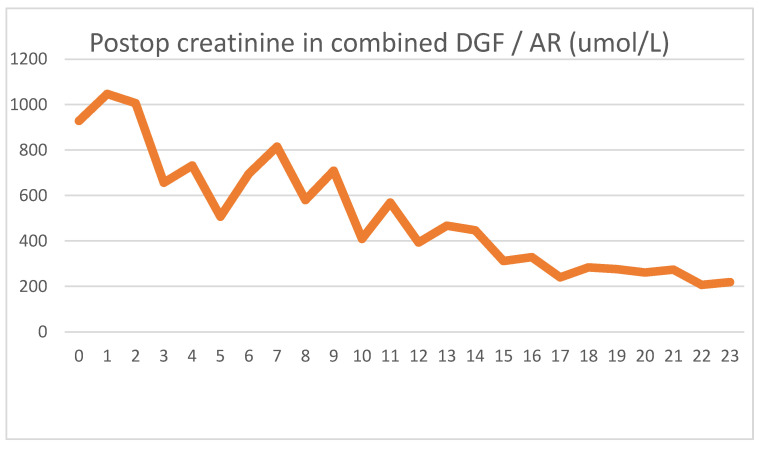
Daily creatinine values showing the effects of both HD treatments and partial graft function.

**Figure 8:**
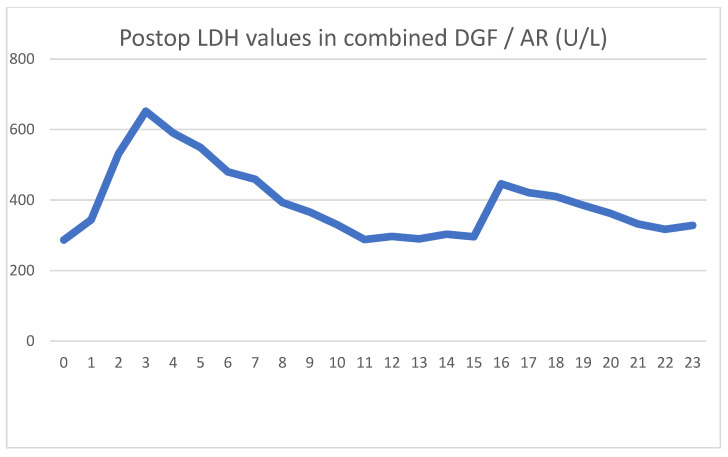
Daily LDH values showing typical spike and a second spike associated with blood transfusion. AR did not have an effect on LDH levels.

She was implanted with a DBD graft, but her biopsy showed major preservation damage to the tubules (ATN II). No immediate post-implantation graft function was observed. Her postoperative lab values were as follows Figure 7 and Figure 8.

After showing minor signs of graft function, her urine output ceased on the second postoperative day. The intraoperative biopsy result, combined with a negative US exam, led us to believe that ischemic damage was to blame for the loss of function. Parallel to the use of HD, the graft function returned, and we were able to suspend HD treatments. We used US examinations several times, but never saw signs of acute rejection. On day 16, we transfused red blood cells to treat her anemia, and again, a transitory serum LDH spike was seen.

Because of the unsatisfactory graft function and declining LDH levels, we performed a biopsy on day 18, the result of which showed pathological signs of acute rejection. We started steroid therapy, and her serum creatinine levels began to decline. Her daily urine output stabilized around 2000 mL/day, with her creatinine level being 211 umol/L at discharge.

During follow-up, we had to apply a course of anti-rejection therapy one more time. Months later, the patient was diagnosed with a small bowel obstruction, which was solved operatively. In the 2 years since transplantation, her creatinine levels have continued to steadily decline toward normal values.

As usual, we saw the postoperative LDH increase, which did not peak on day 1, but continued to rise for three days. The typical HD creatinine pattern could be seen until HD was discontinued on day 11. Meanwhile, the serum LDH levels showed a steady declining trend, again contrary to the expected patterns. The blood transfusion on day 16 again showed an increase in serum LDH, but the graft biopsy on day 18 caused no LDH spike. The acute rejection did not cause increased serum LDH levels. Five days after blood transfusion, LDH levels again stabilized to normal levels.

## 4. Discussion

Our analysis of these four cases reveals clinical patterns not based on the range of LDH values seen, but in the trends observed.

Three of our patients used HD on admission, and one used peritoneal dialysis. Contrary to expectations, the baseline LDH values were near the normal range (300–500 U/L) for every patient regardless of dialysis modality. We expected LDH levels to continually decline as the effects of ESRD and HD wore off after kidney function was restored. This would have likely dominated the LDH trends and resulted in a significant bias toward AR. Instead, we can expect a relatively stable baseline on which any effect is apparent. The passing of these effects causes LDH levels to return to baseline, which helps us to monitor the healing of DGF.

The deviations from baseline, and our decisions based on these, can be summarized as:

- A serum LDH spike of uncertain size can be seen in the 2 days following surgery. This can be attributed to the tissue damage which accompanies surgery and the release of LDH from damaged graft tubules, later washed out of the graft by the recipient’s blood flow. The effect is not instantaneous with the return of circulation, seeing as the process of ischemic damage may not culminate before implantation. After implantation, reperfusion injury would again release LDH into the bloodstream.
- Day-to-day serum creatinine and LDH values do not correlate. However, in cases of acceptable creatinine clearance, we do not perform protocol US examinations or make clinical decisions based on LDH kinetics.
- Accompanying normal graft function, serum LDH levels are expected to decline by day 3. A persistent increase thereafter raises suspicion of significant ischemic damage to graft tubules. By this time, the lack of creatinine clearance requires a US examination, which lacks a baseline radiological picture to compare and lacks diagnostic confidence. This is the point in time at which we do not recommend graft biopsy, and instead rely on LDH values to either continue observation or start therapy.
- As opposed to ischemic damage, acute rejection is not accompanied by an increase in serum LDH. In one case, LDH even sharply decreased in the days before rejection was diagnosed. The mechanism for this decline is not apparent.
- The use of hemodialysis in our patients did not affect serum LDH levels. This is contrary to previous literature, but may be explained by advances in dialysis technology since that study’s year of publication (1990). Again, this is extremely useful, as any significant LDH change due to dialysis would mask other trends. This effect coupled with the postoperative LDH spike would mean that any patient with a primary non-functioning graft would experience confounding factors throughout the whole period of rejuvenation.
- Packed blood cell transfusion causes a significant LDH increase, which takes 2–3 days to normalize. If transfusion happens concurrently with a decline in graft function, caution is needed before assuming ischemic DGF. In our practice, we have since begun to disregard LDH elevation for 2 days after any volume of blood transfusion.

In summary, the diagnostic use of serum LDH in the early post-kidney transplantation period is far from foolproof, although still useful if applied with caution. The intuitive notion that absolute LDH values would correspond with patient outcomes has been previously challenged by Koyama et al. [6], who found only a weak correlation. Nevertheless, we believe that the information to be gleaned from relative LDH changes is being glossed over. Examinations of changes in LDH in cases of rising creatinine levels (without the use of HD) increase clinicians’ confidence at critical times. This lack of confidence often results in delays in therapy or the use of biopsies, which we believe could be avoided.

We theorize that a persistent trend of LDH increasing after transplantation indicates graft preservation damage and raises confidence in a diagnosis of ischemic DGF. In this case, we generally adopt a conservative approach.

A postoperative creatinine rise, not accompanied by an LDH rise, raises suspicion of acute rejection, and we generally repeat US examinations daily. The time needed to acquire laboratory information is virtually the same as that for a US examination, while being faster and safer than a graft biopsy. As such, we believe that serum LDH kinetics can be useful to decide the need for and timing of repeated US examinations, and more importantly, graft biopsy. In cases where both the clinical picture and US are not useful, we initiate anti-rejection therapy based on LDH kinetics.

As our case series lacked statistical analysis, we welcome the publication of a prospective study using a large cohort. A prerequisite of such a study would be meticulous daily documentation of LDH, clinical events, diagnostic results, and confounding factors. 

## Data Availability

The data presented in this study are available upon request from the corresponding author. The data are not publicly available due to local HIS protocols.
